# A Novel ELISA-Based Peptide Biosensor Assay for Screening ABL1 Activity *in vitro*: A Challenge for Precision Therapy in BCR-ABL1 and BCR-ABL1 Like Leukemias

**DOI:** 10.3389/fphar.2021.749361

**Published:** 2021-11-19

**Authors:** Oksana Montecchini, Stefania Braidotti, Raffaella Franca, Giulia Zudeh, Christian Boni, Claudio Sorio, Eleonora Toffoletti, Marco Rabusin, Alberto Tommasini, Giuliana Decorti, Gabriele Stocco

**Affiliations:** ^1^ Department of Medical, Surgical and Health Sciences, University of Trieste, Trieste, Italy; ^2^ Department of Life Sciences, University of Trieste, Trieste, Italy; ^3^ Department of Medicine, University of Verona, Verona, Italy; ^4^ Division of Hematology and Bone Marrow Transplantation, Azienda Ospedaliero-Universitaria, Udine, Italy; ^5^ Institute for Maternal and Child Health (I.R.C.C.S) Burlo Garofolo, Trieste, Italy

**Keywords:** ABL1-class BCR-ABL1 like acute lymphoblastic leukemia, ABL1 peptide biosensor, *in vitro* ELISA assay, ABL1 tyrosin kinase inhibitors, precision therapy

## Abstract

The pathogenic role of the overactivated ABL1 tyrosine kinase (TK) pathway is well recognized in some forms of *BCR-ABL1* like acute lymphoblastic leukemia (ALL); TK inhibitors represent a useful therapeutic choice in these patients who respond poorly to conventional chemotherapy. Here we report a novel peptide biosensor (P_ABL_)-ELISA assay to investigate ABL1 activity in four immortalized leukemic cell lines with different genetic background. The P_ABL_ sequence comprises an ABL1 tyrosine (Y) phosphorylation site and a targeting sequence that increases the specificity for ABL1; additional peptides (Y-site-mutated (P_ABL_-_F_) and fully-phosphorylated (P_PHOSPHO_-_ABL_) biosensors) were included in the assay. After incubation with whole cell lysates, average P_ABL_ phosphorylation was significantly increased (basal vs. P_ABL_ phosphorylation: 6.84 ± 1.46% vs. 32.44 ± 3.25%, *p*-value < 0.0001, two-way ANOVA, Bonferroni post-test, percentages relative to P_PHOSPHO_-_ABL_ in each cell line). Cell lines expressing ABL1-chimeric proteins (K562, ALL-SIL) presented the higher TK activity on P_ABL_; a lower signal was instead observed for NALM6 and REH (*p* < 0.001 and *p* < 0.05 vs. K562, respectively). Phosphorylation was ABL1-mediated, as demonstrated by the specific inhibition of imatinib (*p* < 0.001 for K562, NALM6, ALL-SIL and *p* < 0.01 for REH) in contrast to ruxolitinib (JAK2-inhibitor), and occurred on the ABL1 Y-site, as demonstrated by P_ABL-F_ whose phosphorylation was comparable to basal levels. In order to validate this novel P_ABL_-ELISA assay on leukemic cells isolated from patient’s bone marrow aspirates, preliminary analysis on blasts derived from an adult affected by chronic myeloid leukaemia (*BCR-ABL1* positive) and a child affected by ALL (*BCR-ABL1* negative) were performed. Phosphorylation of P_ABL_ was specifically inhibited after the incubation of *BCR-ABL1* positive cell lysates with imatinib, but not with ruxolitinib. While requiring further optimization and validation in leukemic blasts to be of clinical interest, the P_ABL_-based ELISA assay provides a novel *in vitro* tool for screening both the aberrant ABL1 activity in *BCR-ABL1* like ALL leukemic cells and their potential response to TK inhibitors.

## Introduction

In clinics, a sensitive, quick, convenient and versatile detection method to measure aberrant kinase activity is desirable for many pathologies, including various forms of leukemias, and could be important to improve diagnosis and treatment. The Abelson (ABL)-1 tyrosine kinase (TK) belongs to the non-receptor TK family and is an ubiquitously expressed cytosolic enzyme that plays a role in many key processes linked to cell growth and survival as well as to cell differentiation, cell adhesion and migration (Greuber et al., 2013). Oncogenic forms of ABL1, in particular the *BCR-ABL1* fusion gene with different breakpoints in *BCR* gene, are well-recognized for their pathogenic role in leukemias, including chronic myeloid leukemia (CML), some forms of acute myeloid leukemia (AML), and acute lymphoblastic leukemia (ALL). *BCR-ABL1* results from the chromosomal translocation t (9;22), and encodes for the chimeric BCR-ABL1 protein that leads to an aberrant costitutive activation of the ABL1 proliferation pathway. In the chimeric BCR-ABL1, the N-terminal BCR region interferes with the negative regulation of ABL1 and mediates dimerization of the protein that autophsophorylates the kinase, fully and constitutively activating it (Greuber et al., 2013). Moreover, in ALL, *BCR-ABL1* like forms exist [10–15% of B-ALL pediatric cases, incidence increased with age (Roberts et al., 2014a)], presenting blasts that are transcriptionally related to *BCR-ABL1* expressing cells althought they lack the t(9;22) transloctation. These forms are recognized as a high-risk ALL subtype across all clinical studies, being characterized by an unfavorable prognosis and a higher relapse rate in comparison to other B-ALL subtypes (Roberts et al., 2014a; Boer et al., 2015). *BCR-ABL1* like cells harbour a multitude of other genomic rearrangements that involves either the ABL encoding genes (e.g., *ABL1, ABL2*), the JAK-kinase genes (e.g., *JAK1, JAK2*) or their upstream receptors (e.g., *PDGFRB, CSF1R. CRLF2, EPOR*) (Roberts et al., 2012; Boer et al., 2017), resulting in the presence of chimeric TK costitutively activating either the ABL1 or the JAK-STAT proliferation pathway (Roberts et al., 2014b; Boer and den Boer, 2017). A third, smaller group is characterized by involvement of genetic fusions that lead to altered TK in the RAS pathways (Ofran and Izraeli, 2017).

In reference to the development of a peptide biosensor to detect ABL1 activity, it is important to consider that ABL1 mediates its catalytic function by protein-protein interactions through three SRC homology domains (highly conserved SH3–SH2–SH1 cassette) located at the ABL1 N-terminal. SH1 binds and cleaves ATP, and mediates tyrosine (Y) phosphorylation in target substrate proteins; SH3 and SH2 domains function as interaction modules for targets and as allosteric inhibitors of the catalytic SH1 domain (Corbi-Verge et al., 2013). The consensus motif of an ABL1 substrate is I/V/-**Y**-X-X-P/F (where X is any amino acid**, Y** representing phosphorylation site). This sequence has been considered as the base to develop a peptide biosensor to detect ABL1 activity and was found by screening a library of billion distinct peptides for interaction with purified ABL1 (Songyang et al., 1994; Songyang et al., 1995). Phospho-peptides generated by the incubation with the TK of interest were isolated and sequenced to determine the optimal aminoacid tolerated at each position. The optimized aminoacidic sequence EAI**Y**AAPFAKK (named as “*reporter*” sequence in this manuscript), was designed. This sequence represents an artificial kinase substrate, not a naturally occurring motif of endogenous proteins (Songyang et al., 1994; Songyang et al., 1995) and corresponds to the commercially available Abltide (P_ABLTIDE_), nowadays largely accepted as a reference peptide biosensor for screening ABL1 activity. However, P_ABLTIDE_ is suitable only for biochemical research use (i.e., enzymatic assays on purified ABL1 or pathogenic chimeric ABL1 proteins), and is not intended for use in clinics and diagnostics because of the lack of serum stability, of cell-penetrating properties and of specific ABL1 targeting among other kinases in primary cells (Wu et al., 2010; Henriques et al., 2013). An additional optimized stretch of amino acids (APTYSPPPPP, named as *“targeting”* in this manuscript) has been designed using biocomputing tools on the basis of physicochemical reasoning, and is recognized by the SH3 domain of ABL1 protein (Pisabarro and Serrano, 1996). This sequence could be linked to the *reporter* region, resulting in a novel artificial peptide (named P_ABL_ in this manuscript) with an increased specificity for ABL1. This peptide was further developed to detect intracellular ABL1 kinase activity in live intact cells (Placzek et al., 2010; Tang et al., 2012; Yang et al., 2013), and was applied to the investigation of the activity of the chimeric BCR-ABL1 in whole cell lysates of patients affected by CML (Yang et al., 2013).

The introduction of targeted therapy with imatinib and other small-molecule inhibitors that target BCR–ABL1, competing at the level of the ATP binding site of the kinase, has significantly improved the outcome in patients with *BCR-ABL1* positive leukemias (An et al., 2010; Biondi et al., 2018). It was thus hypothesized that TKIs specific to ABL1 (e.g., imatinib) or JAK (e.g., ruxolitinib) might be valuable options also for *BCR-ABL1* like ALL cases of the ABL-class or JAK-class respectively (Boer et al., 2017; Fazio et al., 2020), and might represent a better therapeutic choice for these patients, who respond poorly to conventional chemotherapy (Den Boer et al., 2009; Roberts et al., 2012; Boer et al., 2015). Nowadays, several ongoing studies are assessing this issue (clinicaltrials.gov identifiers: NCT02883049, NCT02723994, NCT03117751, NCT02420717, NCT03571321). The heterogeneity in their genetic background makes the identification of *BCR-ABL1* like ALL very challenging. Currently, the molecular analyses carried out on patient’s blasts at diagnosis include karyotyping, fluorescence *in situ* hybridization (FISH) or RT-PCR to detect few specific rearrangements. Useful high-throughput genomic approaches such as exome, whole genome and whole transcriptome sequencing are still too expensive to be routinely employed in clinical practice on large patients’ cohorts, particularly in low-income countries (Boer et al., 2017). However, none of these genetic methods can give functional information about the aberrant kinase activity or about blasts sensitivity to TKI. This highlights the importance of having functional assays for TKs with key pathogenic role rather than genomic characterization as tools of precision therapy in *BCR-ABL1* like ALL patients (Franca et al., 2018). Functional TK assays would represent an important tool for clinicians also for *BCR-ABL1* CML and ALL: they could be used to guide the choice of TKI for the best target therapy and to identify primary TKI resistance in a timely manner at diagnosis. Indeed, both imatinib and dasatinib pose resistance problems in BCR-ABL1 positive patients: ABL1 kinase domain mutations have been detected in 30–90% patients who failed imatinib and in 20–80% of patients who failed dasatinib (Gunnar Cario et al., 2020). Identifying in a timely manner the best TKI to be used at diagnosis or during treatment would optimize the chances of survival in patients, and would overcome the possible issue of primary and acquired TKI resistance.

The aim of this study was to investigate the role of P_ABL_ (*“reporter”* + “*targeting”* sequence) as a biosensor able to quantify the ABL1 phosphorylation activity in whole lysates of immortalized leukemic cell lines, including *BCR-ABL1* like ALL cells. Kinase activity was monitored *in vitro* by an ELISA assay. Long-term goal is to set up a point-of-care device to improve the diagnosis and clinical management of *BCR-ABL1* and *BCR-ABL1* like leukemias in clinics. Far from overcoming the importance of monitoring the minimal residual disease, these devices could nonetheless boost the therapeutic monitoring by measuring the residual TK activity or the acquired resistance to these drugs in residual blasts.

## Materials and Methods

### Drug and Chemicals

Imatinib (CDS022173, Sigma-Aldrich, Italy) was dissolved in DMSO at a concentration of 1 mg/ml (2 mM) and ruxolitinib (11,609 Cayman Chemical, United States) in ethanol at a concentration of 10 mg/ml (32.6 mM), according to manufacturer instructions.

### Cell Cultures

The study was performed on four human leukemia cell lines purchased from the DSMZ GmbH (Germany): NALM6 (ACC 128), ALL-SIL (ACC 511), K562 (ACC 10), REH (ACC 22). Additionally HEL (ACC 11) and SET2 (ACC 608) cell lines, kindly provided by Professor A. Vannucchi (Department of Experimental and Clinical Medicine, University of Florence), were included in MTT cell viability assay.

### Cell Lysates Preparation and Western Blot

Cell protein lysates were prepared for both western blot and P_ABL_-based ELISA assays. The method of extraction has been optimized by Professor Sorio (University of Verona) and requires the use of kinase and phosphatase inhibitors to maintain the integrity of *BCR-ABL1* in the K562 cell line (Supplementary Material). Western blots with antibodies against PDGFRB, ABL1, Phospho-ABL1 (Y245) and actin were performed as described in Supplementary Material.

### Cell Viability Assays

The effect of TKI on NALM6, ALL-SIL, K562, REH, HEL, SET2 cell lines was determined using the 3-(4,5-dimethylthiazol-2-yl)-2,5-diphenyltetrazolium bromide (MTT) assay and trypan blue exclusion assay, as described in Supplementary Material.

### Peptide Biosensors

Biosensor peptides were synthesized by GenScript (Piscataway, NJ08854, United States) with 98% purity, and are shown in Table 1. P_ABL_ comprises two tyrosine, one in the “*target*” and one in the “*reporter*” region. P_ABL-F_ is a site-mutated biosensor with the tyrosine in the “*reporter*” region replaced by phenylalanine, and was used in order to verify the specificity of the phosphorylation signal on the catalytic site. P_PHOSPHO-ABL_ is a fully phosphorylated version of P_ABL_, used for relative quantification of the phosphorylation level obtained.

**TABLE 1:** Biosensor peptides employed in ELISA assay, their sequence and molecular weight. The “*reporter*” sequence is shown in italic, and the “*targeting*” sequence is underlined. In the phosphorylation site of ABL1, tyrosine and peptide modifications are shown in bold.

Peptides	Sequence	Molecular weight
**P**_**ABL**_	*EAI* ***Y*** *AAPFAKK*{Lys(biotin)}GGCGGAPTYSPPPPPG	2,956.41 Da
**P**_**ABL-F**_	*EAI* ***F*** *AAPFAKK*{Lys(biotin)}GGCGGAPTYSPPPPPG	2,940.41 Da
**P**_**PHOSPHO-ABL**_	*EAI* ***Y*** (***P***)*AAPFAKK*{Lys(biotin)}GGCGGAPTYSPPPPPG	3,036.39 Da
**P**_**ABLTIDE**_	*EAI* ***Y*** *AAPFAKK*{Lys(biotin)}	1,562.90 Da

### P_ABL_-Based ELISA Assay

**FIGURE 1:**
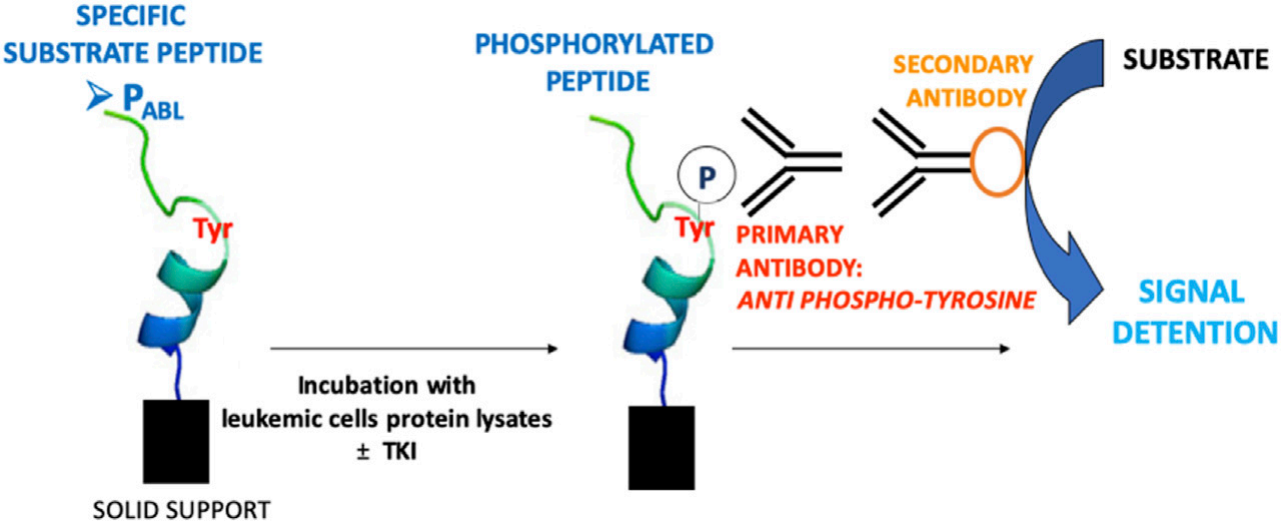
Schematic illustration of P_ABL_-based ELISA assay principle.

The P_ABL_-based ELISA assay procedure is shown in Figure 1. All biosensor peptides contain a biotin tag that allows their anchoring to a neutravidin-coated plate (786-766, G-Bioscience, United States), when loaded onto the plate at a concentration 0.5 µM in PBS and shacked for 1 h at room temperature (RT). To avoid non specific signals the plate was first washed with a Quencher Buffer (100 μl/well, PBS +0.1% Tween 20 pH 7.4 + 0.4% BSA) for 20 min while shaking. 4 μg of cell lysates were then loaded into the plate and incubated in Tyrosine Kinase Buffer (4 mM Tris-HCl pH 7.5, 10 mM MgCl_2_, 0.1 mM EDTA, 0.01% TritonX) with 100 μM ATP, 1x Roche Inhibitors, 2 mM DTT, 0.1 mM Na_3_VO_4_ and MilliQ-water (final volume 100 μl) for 1 h at RT. To evaluate the effect of TKI, cell lysates were pre-incubated for 15 min at RT with drugs (imatinib 5 μM, ruxolitinib 52 nM e 5 μM, chosen accordingly to plasma steady state concentrations in patients) before adding on the biosensor-coated plate. The phosphorylation of the peptides was measured using an anti phosphorylated tyrosine antibody (05-1050 4G10 Platinum, EDM Millipore Corporation, dilution 1:10,000 in Quencher Buffer, 1 h, RT while shaking) and detected with a secondary antibody ECL Anti Mouse IgG, HRP from goat (5210-0159, Sera-care, United States, dilution of 1:6,000 in Quencher Buffer, 1 h, RT while shaking). Signal was allowed by adding a solution of citrate buffer pH 6.0, 3% H_2_O_2_, 0.5% Amplex Ultra Reagent Invitrogen (A36006, Thermofischer Scientific, Italy) and measured 25 times every 0.2 s (544 nm excitation, 590 nm emission wavelengths). P_PHOSPHO-ABL_ was included in each experiment and added to lysates of any cell lines used as an ELISA assay positive control and as a tool to measure the maximal fluorescent signal achievable on the biosensor. P_ABL_ and P_ABL-F_ phosphorylation levels were expressed as a percentage related to P_PHOSPHO-ABL_ phosphorylation in the same cell line, according to the following formula: (mean P_ABL_)/(mean P_PHOSPHO-ABL_) × 100.

### Purification of Primary Mononuclear Cells From Patients

Two patients were included in this study. The first was a 40–50 years old male adult affected by chronic myeloid leukaemia (p210 BCR-ABL1 positive), treated at Azienda Ospedaliero-Universitaria in Udine (Italy). The second was a 1–3 years old female child (affected by *BCR-ABL1* negative ALL, data regarding *BCR-ABL1* like translocations not known) treated at IRCCS Burlo Garofolo in Trieste (Italy). Bone marrow aspirates were collected at onset as part of diagnostic procedures, and used for research purpose only when clinical procedures had been completed. Patients’ bone marrow aspirates (∼3–5 ml) were diluted with PBS to a final volume of 10 ml and loaded on Ficoll-PaqueTM Plus (5 ml); after centrifugation (800 xg, 20 min, 15°C), interphase was recovered and washed twice with PBS (7 ml, 300 xg, 10 min, 15°C); 20 × 10^6^ mononuclear cells were used for cell lysate preparation.

### Statistical Analysis

Data obtained from the MTT assay were analyzed using a nonlinear regression on GraphPad Prism8; IC_50_ was determined from the dose-response curve. Statistical analysis was performed using two-way ANOVA and Bonferroni multiple comparison test; statistical significance was set at *p* < 0.05.

## Results

### Cell Lines Characterization

Four human leukemia cell lines with different genetic background were selected and initially characterized by western blot to confirm the presence of candidate chimeric proteins: NALM6 and ALL-SIL cells were chosen because they harbor the gene fusions *ETV6-PDGFRB* (Matheson and Hall, 2003) and *NUP214-ABL1* respectively, both belonging to the *BCR-ABL1* like aberrations and both leading to ABL1 pathway overactivation (Zhou and Yang, 2014). Because of the t(9;22) rearrangement encoding BCR-ABL1, K562 was used as control model for the P_ABL_-based ELISA assay. REH cells are carriers of the *ETV6-RUNX1* (*TEL-AML1*) gene fusion.

**FIGURE 2:**
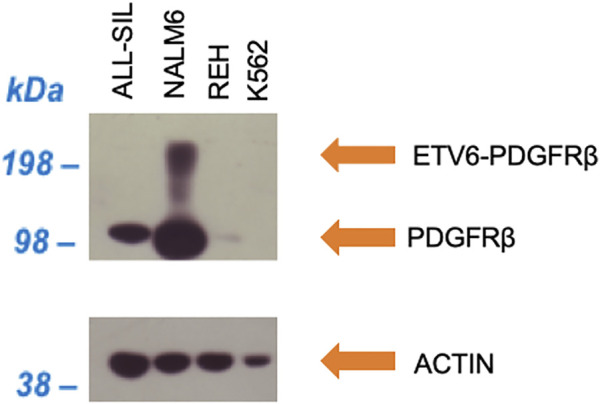
Western blot against human PDGFRB (upper panel) and human actin (lower panel) as loading control.

**FIGURE 3:**
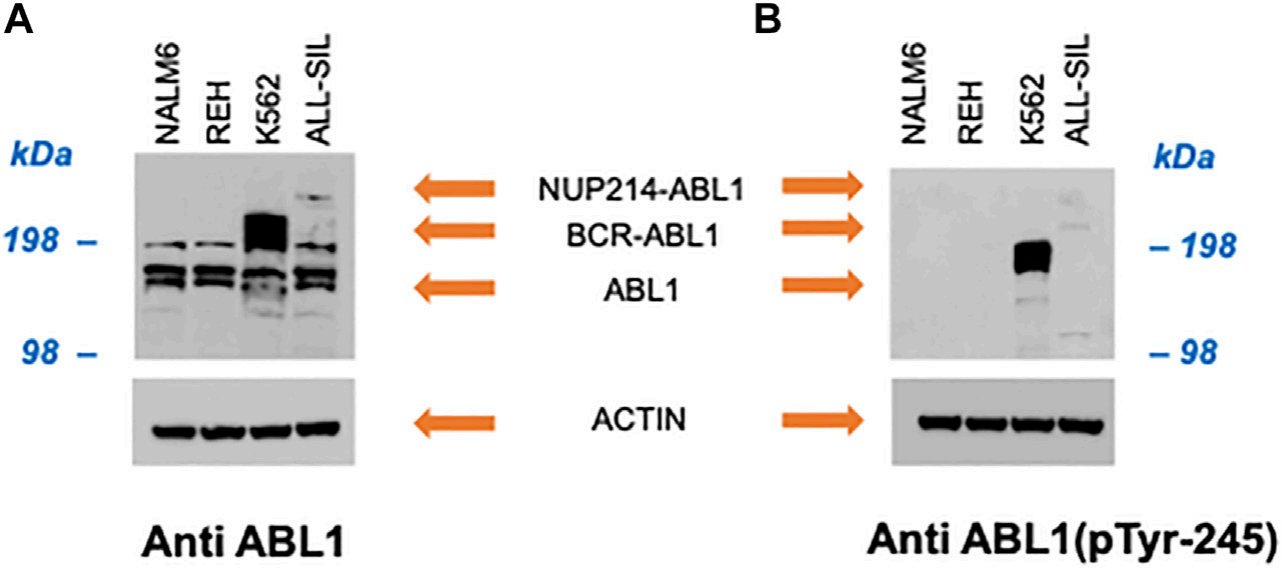
Western blot with **(A)** anti human ABL1 and **(B)** anti human phospho-ABL1. Actin was used as loading control.

In NALM6 the presence of ETV6-PDGFRB was investigated using an antibody directed against human PDGFRB. As showed in Figure 2, a band with molecular weight of ∼100 kDa, corresponding to PDGFRB (predicted molecular weight 125 kDa), was clearly visible in NALM6 and also in ALL-SIL and to a lesser extent in REH cells, while it was lacking in K562. ETV6-PDGFRB has a predicted molecular mass of 76 kDa (Carroll et al., 1996): the monomer should migrate to an apparent molecular weight of 90–100 kDa in SDS-PAGE. In NALM6, a specific band >200 kDa appeared, corresponding to the presence of oligomeric and multimeric complexes of the chimeric protein. Western blots using antibodies against ABL1 and phospho-ABL1 (pTyr-245) were performed as shown in Figure 3. In K562 cells the expected band around 210 kDa, corresponding to BCR-ABL1, was visible and in ALL-SIL cells a band around 300 kDa, corresponding to the chimeric protein NUP214-ABL1, was also evident (Figure 3A). Wild type ABL1 was visible in NALM6, REH, K562, ALL-SIL in comparable amount (Supplementary Figure S1). Western blot with anti phospho-ABL1 showed a band of 210 kDa, corresponding to phosphorylated BCR-ABL1 protein and a band at lower molecular weight (around 125 kDa), corresponding to phosphorylated ABL1, in K562 cells (Figure 3B). Phospho-proteins were not detected in other cell lines.

**FIGURE 4:**
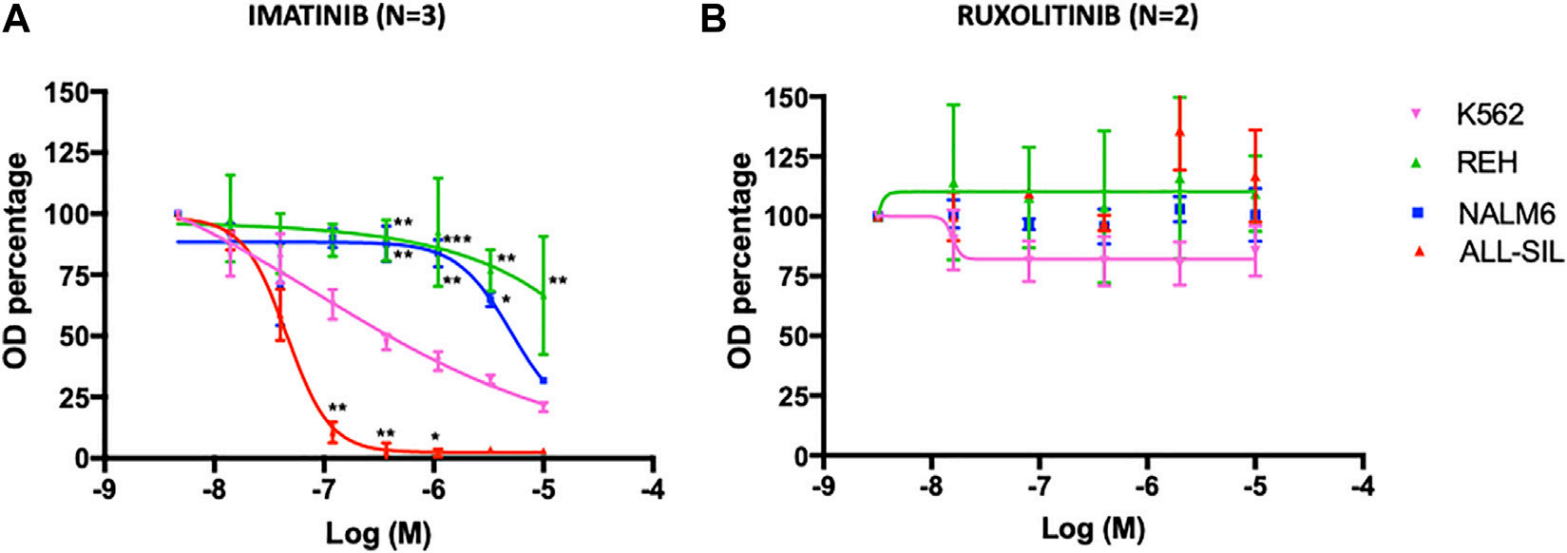
Dose-response curve with **(A)** imatinib and **(B)** ruxolitinib. Leukemia cell lines were seeded at 12,000 cells/well with a range of concentrations of 0.014–10 µM for imatinib and 0.016–10 µM for ruxolitinib. MTT assay was performed after 72 h of incubation. Error bars represent mean ± SEM (*n* = 3 for imatinib, *n* = 2 for ruxolitinib). REH, NALM6 or ALL-SIL versus K562: *p < 0.05; **p < 0.001, ***p < 0.0001 two-way ANOVA, Bonferroni post-test.

The cytotoxic effect of TKI (imatinib and ruxolitinib) on cell lines was determined using the MTT assay (Figure 4). Viability of the 2 cell lines harboring the *ABL1* fusion genes was clearly impaired by imatinib, and differed significantly each other (*p* < 0.0001) with the ALL-SIL being more sensitive (IC_50_ = 46.9 ± 6.99 nM) than K562 [0.38 ± 0.13 μM in accordance to previously reported values (Quintás-Cardama and Cortes, 2009)]. Imatinib affected also NALM6 viability at higher concentration (IC_50_ = 5.56 ± 0.65 μM), whereas a survival of ∼70% was observed in REH at 10 μM, the highest concentration tested (Figure 4A). As expected, cell lines were resistant to ruxolitinib, a specific JAK1/2 inhibitor (Figure 4B), in contrast to what was observed for HEL (IC_50_ = 1.19 ± 0.33 μM) and SET2 cells (IC_50_ = 48.58 ± 0.56 nM), used as positive control (Supplementary Figure S2). Cytotoxic effects evaluated by the MTT assay could be confounded by metabolically inactive cells. Therefore, cell viability was also confirmed by trypan blue exclusion assay (Supplementary Table S1). Imatinib concentrations were chosen according to MTT results, being 0.38 µM the K562 IC_50_, 5.5 µM the NALM6 IC_50_ and 10 µM the highest drug concentration tested. Comparable but not perfectly overlapping values in survival rates was observed for K562 at 0.38 µM (37.53 ± 9.11% vs. the expected 50% calculated by MTT results measuring mitochondrial cell activity) and for ALL-SIL (29.30 ± 12.86% at 0.38 µM and 18.58 ± 3.59% at 10 µM vs. the almost 100% mortality reached in MTT assays). A good correspondence was instead observed for NALM6 and REH (survival rates 51.17 ± 9.93% at 5.5 µM and 74.68 ± 31.53% at 10 μM, respectively). In MTT assay, cells were resistant to ruxolitinib at concentration lower than 10 μM, therefore this concentration and a higher one (i.e., 50 µM) were used for trypan blue exclusion assay. As expected, cell viability was only slightly affected at ruxolitinib concentration of 10 µM and required exposure to very high concentration of the drug, well outside the therapeutic range of clinical interest, to be compromised.

### Peptide Biosensor *in vitro* Functional Analysis

**FIGURE 5:**
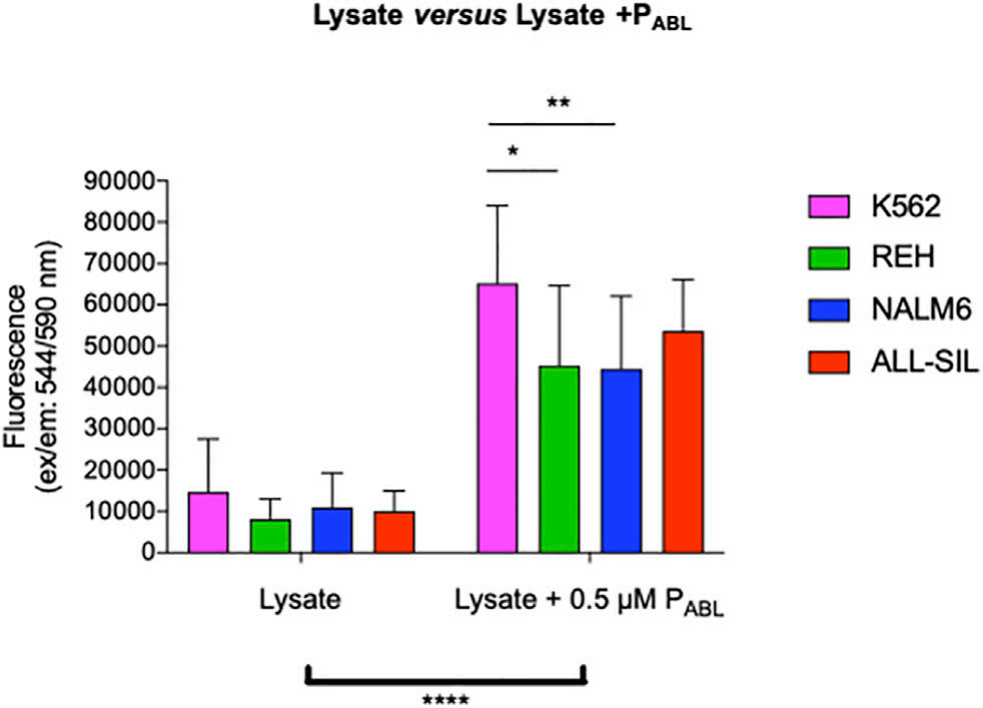
P_ABL_-based ELISA assay. The graph shows data obtained from 12 independent experiments for K562, REH and NALM6 cell lines and seven experiments for the ALL-SIL cell line. Fluorescence values in ordinate. There is a significant increase in terms of phosphorylation levels for all the lines after incubation with the peptide P_ABL_ (****, *p*-value ANOVA two-way 0.0001). K562 line showed the highest signal, significantly higher compared to the NALM6 and REH (*p*-value ANOVA two-way Bonferroni post-test for multiple comparison: *: 0.05; **: 0.001).

**FIGURE 6:**
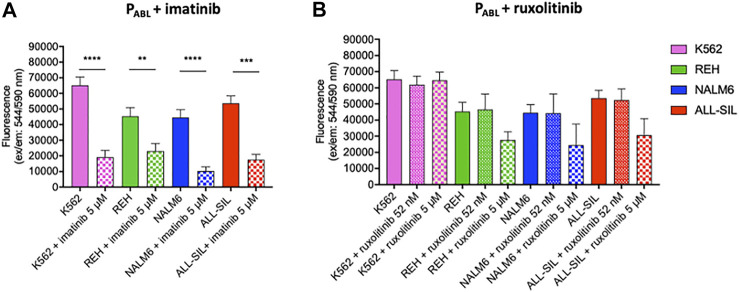
P_ABL_-based ELISA assay in the presence of **(A)** imatinib and **(B)** ruxolitinib. Fluorescence values in ordinate. Imatinib determines a significant decrease of P_ABL_-phosphorylation in all cell lines. The reduction is particularly evident for those cell lines with alterations affecting the ABL1-pathway (K562, and ALL-SIL) and is less pronounced in REH. Ruxolitinib treatment does not affect P_ABL_-phosphorylation. *p*-value according to two-way ANOVA, Bonferroni post-test for multiple comparison: ****, <0.0001; ***, <0.001; **, <0.01.

The P_ABL_-based ELISA analysis showed a significant phosphorylation of the P_ABL_ biosensor probe after incubation with all four leukemic cell lines tested (two-way ANOVA, Bonferroni post-test, *p*-value < 0.0001, Figure 5). Supplementary Table S2 reports results of the phosphorylated P_ABL_ as percentages relative to the fully phosphorylated P_PHOSPHO-ABL_, used as positive reference value in each cell line: average basal phosphorylation of lysates was 6.84 ± 1.46% in absence of biosensor vs. 32.44 ± 3.25% in presence of P_ABL._ As shown in Figure 5, K562 cells presented the highest phosphorylation level (mean fluorescence intensity (FI): 65,185.81), similar to the P_ABL_ phosphorylation obtained after incubation with ALL-SIL lysates (mean FI: 53,671.62). A lower signal was instead observed for NALM6 (mean FI: 44,495.64, *p*-value < 0.01 vs. K562) and REH (mean FI: 45,352.60, *p*-value < 0.05 vs. K562). To confirm the contribution of ABL1 activity on the probe, cell lysates were pre-incubated with ABL1 specific and non-specific inhibitor (imatinib and ruxolitinib, respectively). Pre-incubation of lysates with 5 μM imatinib lead to a significant decrease of P_ABL_ phosphorylation (two-way ANOVA, Bonferroni post-test, *p*-value < 0.0001, Figure 6A). In contrast, ruxolitinib (a JAK1/2 specific inhibitor) did not affect significantly the P_ABL_ phosphorylation neither at 52 nM nor at 5 μM, although a slight decrease in P_ABL_ signals was observed in NALM6, REH and ALL-SIL at the highest concentration likely due to an aspecific effect (Figure 6B).

An ELISA assay was performed on K562 lysates to compare the P_ABL_ (“*target*” + “*reporter*” sequences) to P_ABLTIDE_ (“*reporter*” sequence only). P_ABL_ shows a higher phosphorylation compared to P_ABLTIDE_ (two-way ANOVA, Bonferroni post-test, *p*-value 0.023), with a significant decrease after imatinib pre-treatment of lysates (*p*-value 0.0027, Supplementary Figure S3A). Interestingly, no significant changes in phosphorylation level of P_ABLTIDE_ was observed increasing both the amount of lysate and the probe concentration (Supplementary Figure S3B).

P_ABL_ comprises two tyrosines, one in the “*target*” and one in the “*reporter*” region. In order to verify the specificity of the phosphorylation signal on the catalytic site, a site-mutated biosensor was introduced in the assay (i.e., P_ABL-F_, with the tyrosine in the “*reporter*” region replaced by phenylalanine). The levels of P_ABL-F_ phosphorylation were similar among cell lysates, and did not differ from the basal fluorescence signals detectable in all cell lines in the absence of peptide (Supplementary Figure S4 and Supplementary Table S3).

**FIGURE 7:**
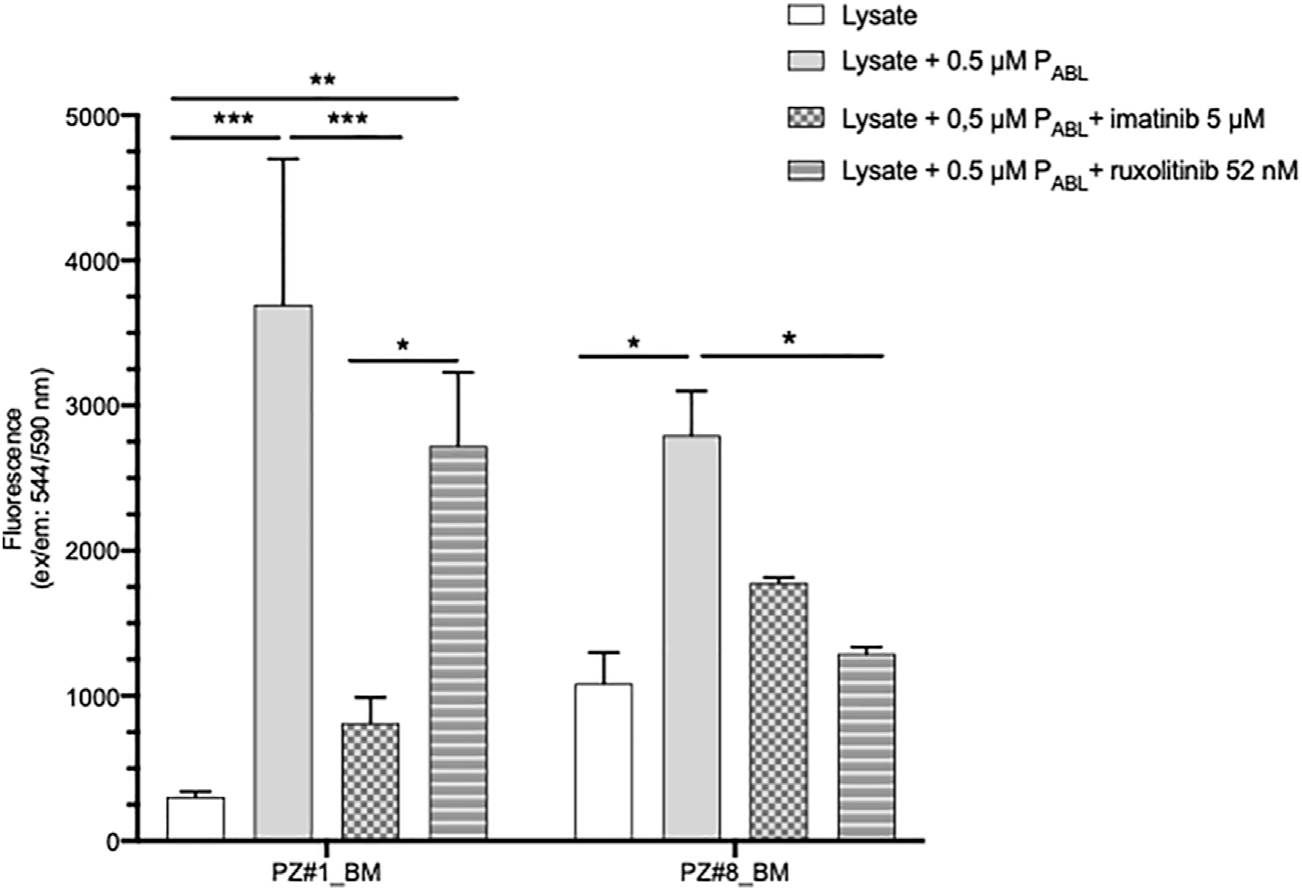
P_ABL_-based ELISA assay in patients’ primary cells lysates. Pt#1 is an adult male patient affected by CML (p210 BCR-ABL1 positive); Pt#2 is a female child affected by *BCR-ABL1* negative ALL (*BCR-ABL1 like* translocations not known). Fluorescence values in ordinate. *p*-value according to two-way ANOVA, Bonferroni post-test for multiple comparison: ***, <0.001; **, <0.01, * <0.05.

To further validate the novel P_ABL_-based ELISA assay, lysates of primary leukemic cells derived from two patients, one affected by chronic myeloid leukaemia (p210 *BCR-ABL1* positive, Pt#1) and another by ALL (*BCR-ABL1* negative, Pt#2), were also used. In both cases, the P_ABL_ biosensor probe was phosphorylated after incubation with cell lysates (two-way ANOVA, Bonferroni post-test, Pt#1: *p*-value < 0.001, Pt#2: *p*-value < 0.05, Figure 7). However, inter-patient differences were observed in the presence of TKI. The pattern of drug inhibition was compatible to the expected ABL1-mediated P_ABL_ phosphorylation in *BCR-ABL1* positive pt#1 (P_ABL_ signal inhibited by imatinib (*p*-value < 0.001) but not by ruxolitinib), in contrast to what was observed in *BCR-ABL1* negative pt#2 (P_ABL_ signal inhibited by ruxolitinib (*p*-value < 0.05) but not by imatinib). Cells sensitivity to imatinib and ruxolitinib could not be assessed due to the scanty biological material available.

## Discussion

In recent years, several potential peptides allowing the study of kinases’ activity *in vitro* have been developed (Lipchik et al., 2015; Perez et al., 2019). The artificial peptide P_ABL_ resembles the previously published cell-permeable biosensor peptide used to monitor the BCR-ABL1 function in CML (Yang et al., 2013), with “*reporter*” and “*target*” sequences in common. Here, P_ABL_ was employed to validate its use in lysates of ALL cell lines with different genetic background, including those harboring *BCR-ABL1* like ALL translocations of the ABL-class. The phosphorylation of the P_ABL_ probe was detected after the incubation with cell lysates. Interestingly, none of the cell lysates induced an increase of P_ABL-F_ phosphorylation, indicating a kinase discriminatory action on the tyrosine in the “*reporter*” region. K562 cells showed the highest fluorescence signal on P_ABL_ although comparable to that observed for ALL-SIL. Both these cell lines strongly rely on ABL1 for survival and proliferation, as demonstrated by MTT and trypan blue viability assays, although only BCR-ABL1 and not NUP214-ABL1 could be detected in the phosphorylated form by western blot. It is known that NUP214-ABL1 displays much lower auto-phosphorylation than BCR-ABL1 (Brasher and Van Etten, 2000; De Keersmaecker et al., 2008a; De Keersmaecker et al., 2008b). Because of this lower activation, ALL-SIL cells are more sensitive than K562 to imatinib, an ABL1 inhibitor that targets specifically the inactive conformation of the ABL1 kinase. Nonetheless, BCR-ABL1 and NUP214-ABL1 largely overlap in their substrate specificity as demonstrated using a peptide array (De Keersmaecker et al., 2008a).

NALM6 cells were chosen because they harbor the gene fusion *ETV6-PDGFRB*. PDRGFB is a cell surface TK receptor that activates the ABL1 pathway (Plattner et al., 2004), and *PDRGFB* rearrangements (*ETV6-PDGRFB* and *EBF1-PDGFRB*) are present in about 1% of *BCR-ABL1* like positive patients (Boer et al., 2017). Recent studies observed a reduction in signalling with imatinib in cases harboring ABL1-class rearrangments (Roberts et al., 2017), and a case-report on a refractory *EBF1-PDGFRB* ALL patient showed the complete remission after the addition of imatinib to the conventional chemiotherapy (Weston et al., 2013). The chimeric protein ETV6-PDGFRB contains the amino-terminal 154 amino acids of ETV6 fused to the transmembrane and cytoplasmic domains of PDGFRB, and has a calculated molecular mass of 76 kDa (Carroll et al., 1996). Western blot using an anti human PDGFRB confirmed the presence of ETV6-PDGFRB in NALM6: the monomer migrated at 90–100 kDa in SDS gel-electrophoresis; however, ETV6-PDGFRB appeared in NALM6 also as oligomeric or multimeric complexes resulting in a band around 200 kDa, as already observed in literature (Sjöblom et al., 1999). The *wild type* PDGFRB was also detectable in all the cell lines (a band around 100 kDa) except for K562. Some investigators report that NALM6 cell line harbors a different translocation, in particular DUX4-rearranged [t (4;14) (q35;q32)]/ERG deletion (Yasuda et al., 2016; Tanaka et al., 2018); however, both our western blotting analysis and cytogenetic information on the t(5;12)(q33.2;p13.2) translocation confirm the presence of the *ETV6-PDGFRB* in the NALM6 cells used in this paper (Wlodarska et al., 1997)^1^. Since PDGRFB is linked to ABL1 activation (Plattner et al., 2003), we expected higher P_ABL_ phosphorylation values also for the *BCR-ABL1* like cell line NALM6, in particular in comparison to REH cells that should not present constitutively active ABL1 based on their genetic profile, but surprisingly, these two cell lines showed similar fluorescence levels. The reason is still unclear, but can be related to the ubiquitous presence of ABL1 in the cell lines (Greuber et al., 2013). Indeed, Western blot analysis with anti-human ABL1 showed the presence of non-phosphorylated ABL1 in both REH and NALM6, without detecting the phosphorylated form.

The specificity of the P_ABL_ phosphorylation signal was also confirmed after treatment with TKI. Indeed, the ABL1-inhibitor imatinib was used at a concentration of 5 μM that, as reported in the literature, represents the steady state plasma concentration after 5–7 days of treatment in adult CML at a dose of 400 mg/day (Druker et al., 2001), and is 20 times higher than the IC_50_ calculated *in vitro* on cellular tyrosine phosphorylation assay (Deininger et al., 2005). At this concentration, a significant decrease in P_ABL_ phosphorylation levels after the incubation with the drug was observed for all cell lines. It is known from the literature that imatinib has an unusually high selectivity to ABL1 because it targets the inactive conformation which is unique to this kinase (Lee and Wang, 2009). Mass spectrometry analysis investigating imatinib-associated proteins in K562 lysates confirmed the direct interaction of the drug with only few targets, including the known interactor BCR-ABL1 and the ABL-releted gene (ARG), Discoidin Domain Receptor Tyrosine Kinase 1 (DDR1) and the proto-oncogene receptor tyrosine kinase (KIT) (Bantscheff et al., 2007; Rix et al., 2007). DDR1 and KIT are receptor tyrosine kinases and their contribution to the ABL1 activation likely depends on extracellular stimuli. Therefore, considering that our *in vitro* system works on cell lysates rather than whole cells in the absence of specific ligands, their confounding contribution to the P_ABL_ phosphorylation, if any, is likely less important. In contrast, contribution of ARG (closely related to ABL1) could be an issue. However, the P_ABL_ *target* region should guarantee an increased specificity for ABL1 compared to ARG. Mass spectrometry studies identified additionally one off-target (non-tyrosine kinase) of imatinib, the NAD(P)H:quinone oxidoreductase NQO2, whose enzymatic function should not influence the peptide phosphorylation (Bantscheff et al., 2007; Rix et al., 2007). In contrast to imatinib, the anti-JAK inhibitor ruxolitinib did not affect the P_ABL_ phosphorylation levels at any of the concentrations selected (52 nM and 5 μM). The concentration of 52 nM was chosen in accordance to pharmacokinetic parameters measured in healthy subjects at steady state following a twice-daily administration of ruxolitinib at 15 mg and is 20 times higher than the IC_50_ of 2.8 ± 1.2 nM calculated *in vitro* for JAK2 by a biochemical enzymatic inhibitory assay (Quintás-Cardama et al., 2010). The second concentration of 5 μM is much higher than the pharmacological range and was included into the P_ABL_-based ELISA assay in order to verify the absence of inhibition, even at this high concentration.

In our *in vitro* ELISA assay, P_ABLTIDE_ was clearly less performing than P_ABL_, designed to increase the specificity to ABL1 among other kinases. Besides the increased specificity of P_ABL_ over P_ABLTIDE_, the ELISA assay proposed in this paper presents other advantages over conventional kinase assay. Firstly, the biosensor phosphorylation was quantified on a solid-phase through a neutravidin-coated plate that allows the binding of the biotin in the P_ABL_ sequence. This high specific binding greatly reduces background noise signals. Secondly, whole cell lysates can be used, avoiding tricky ABL1 purification and bypassing the problems related to the peptide penetration into intact cells. Thirdly, a unique primary antibody, i.e., an anti-phosphotyrosine, is required. This advantage is particularly important for *BCR-ABL1* like ALL because the ELISA assay conditions could be optimized regardless of the specific chimeric protein encoded by the genetic abnormalities in *BCR-ABL* like leukemic cells of the ABL-class.

Initial attempts to validate the novel P_ABL_-based ELISA assay on lysates of patients’ leukemic cells were performed. Although results are preliminary and limited to only two samples, they allow some considerations, to be confirmed by further investigations. As observed in immortalized cell lines, P_ABL_ becomes phosphorylated after the incubation with leukemic cell lysates regardless the presence of a BCR-ABL1 chimeric protein; however, in contrast to them, this phosphorylation is ABL-1 mediated only in some patients in which it is specifically inhibited by imatinib. A different contribution of ABL1 and other TK on P_ABL_ could be hypothesized among different patients, according to their pathogenetic profile.

Taken together, these observations suggest that the P_ABL_-based ELISA assay is suitable for measuring the ABL1 kinase activity of cell lysates through the P_ABL_-Y phosphorylation in the *“reporter*” region. P_ABL_ could be more suitable in detecting aberrant sustained kinase activity of ABL1-chimeric proteins rather than an over-activation of native ABL1 due to upstream signaling; however, a limitation of this study is that the difference between basal and sustained ABL1 activity is still too tiny for the P_ABL_-based ELISA assay to be of practical clinical interest without any further optimization. To confirm its utility, our system should be improved in sensitivity and accuracy, and results could be strengthened by other TKI, the use of different biosensors (e.g., of downstream signaling components such as STAT5 that is known to be activated by ABL1-class rearrangements), and the test of patient samples of known genotype. Nonetheless, the results here described represents the first step towards the setup of a point-of-care device for diagnosis and therapeutic drug monitoring of pediatric *BCR-ABL1* like patients.

## Data Availability

The raw data supporting the conclusion of this article will be made available by the authors, without undue reservation.

## Footnotes

1
https://www.dsmz.de/collection/catalogue/details/culture/ACC-128.

## References

[B1] An X. Tiwari A. K. Sun Y. Ding P. R. Ashby C. R. Jr. Chen Z. S. (2010). BCR-ABL Tyrosine Kinase Inhibitors in the Treatment of Philadelphia Chromosome Positive Chronic Myeloid Leukemia: a Review. Leuk. Res. 34 (10), 1255–1268. 10.1016/j.leukres.2010.04.016 20537386

[B2] Bantscheff M. Eberhard D. Abraham Y. Bastuck S. Boesche M. Hobson S. (2007). Quantitative Chemical Proteomics Reveals Mechanisms of Action of Clinical ABL Kinase Inhibitors. Nat. Biotechnol. 25 (9), 1035–1044. 10.1038/nbt1328 17721511

[B3] Biondi A. Gandemer V. De Lorenzo P. Cario G. Campbell M. Castor A. (2018). Imatinib Treatment of Paediatric Philadelphia Chromosome-Positive Acute Lymphoblastic Leukaemia (EsPhALL2010): a Prospective, Intergroup, Open-Label, Single-Arm Clinical Trial. Lancet Haematol. 5 (12), e641–e652. 10.1016/s2352-3026(18)30173-x 30501871

[B4] Boer J. M. den Boer M. L. (2017). BCR-ABL1-like Acute Lymphoblastic Leukaemia: From Bench to Bedside. Eur. J. Cancer 82, 203–218. 10.1016/j.ejca.2017.06.012 28709134

[B5] Boer J. M. Marchante J. R. Evans W. E. Horstmann M. A. Escherich G. Pieters R. (2015). BCR-ABL1-like Cases in Pediatric Acute Lymphoblastic Leukemia: a Comparison between DCOG/Erasmus MC and COG/St. Jude Signatures. Haematologica 100 (9), e354–7. 10.3324/haematol.2015.124941 26045294 PMC4800707

[B6] Boer J. M. Steeghs E. M. Marchante J. R. Boeree A. Beaudoin J. J. Beverloo H. B. (2017). Tyrosine Kinase Fusion Genes in Pediatric BCR-ABL1-like Acute Lymphoblastic Leukemia. Oncotarget 8 (3), 4618–4628. 10.18632/oncotarget.13492 27894077 PMC5354859

[B7] Brasher B. B. Van Etten R. A. (2000). c-Abl Has High Intrinsic Tyrosine Kinase Activity that Is Stimulated by Mutation of the Src Homology 3 Domain and by Autophosphorylation at Two Distinct Regulatory Tyrosines. J. Biol. Chem. 275 (45), 35631–35637. 10.1074/jbc.M005401200 10964922

[B8] Carroll M. Tomasson M. H. Barker G. F. Golub T. R. Gilliland D. G. (1996). The TEL/platelet-derived Growth Factor Beta Receptor (PDGF Beta R) Fusion in Chronic Myelomonocytic Leukemia Is a Transforming Protein that Self-Associates and Activates PDGF Beta R Kinase-dependent Signaling Pathways. Proc. Natl. Acad. Sci. U S A. 93 (25), 14845–14850. 10.1073/pnas.93.25.14845 8962143 PMC26224

[B9] Corbi-Verge C. Marinelli F. Zafra-Ruano A. Ruiz-Sanz J. Luque I. Faraldo-Gómez J. D. (2013). Two-state Dynamics of the SH3-SH2 Tandem of Abl Kinase and the Allosteric Role of the N-Cap. Proc. Natl. Acad. Sci. USA 110 (36), E3372–E3380. 10.1073/pnas.1303966110 23959873 PMC3767523

[B10] De Keersmaecker K. Rocnik J. L. Bernad R. Lee B. H. Leeman D. Gielen O. (2008). Kinase Activation and Transformation by NUP214-ABL1 Is Dependent on the Context of the Nuclear Pore. Mol. Cel 31 (1), 134–142. 10.1016/j.molcel.2008.05.005 18614052

[B11] De Keersmaecker K. Versele M. Cools J. Superti-Furga G. Hantschel O. (2008). Intrinsic Differences between the Catalytic Properties of the Oncogenic NUP214-ABL1 and BCR-ABL1 Fusion Protein Kinases. Leukemia 22 (12), 2208–2216. 10.1038/leu.2008.242 18784740

[B12] Deininger M. Buchdunger E. Druker B. J. (2005). The Development of Imatinib as a Therapeutic Agent for Chronic Myeloid Leukemia. Blood 105 (7), 2640–2653. 10.1182/blood-2004-08-3097 15618470

[B13] Den Boer M. L. van Slegtenhorst M. De Menezes R. X. Cheok M. H. Buijs-Gladdines J. G. Peters S. T. (2009). A Subtype of Childhood Acute Lymphoblastic Leukaemia with Poor Treatment Outcome: a Genome-wide Classification Study. Lancet Oncol. 10 (2), 125–134. 10.1016/s1470-2045(08)70339-5 19138562 PMC2707020

[B14] Druker B. J. Talpaz M. Resta D. J. Peng B. Buchdunger E. Ford J. M. (2001). Efficacy and Safety of a Specific Inhibitor of the BCR-ABL Tyrosine Kinase in Chronic Myeloid Leukemia. N. Engl. J. Med. 344 (14), 1031–1037. 10.1056/NEJM200104053441401 11287972

[B15] Fazio F. Barberi W. Cazzaniga G. Fazio G. Messina M. Della Starza I. (2020). Efficacy of Imatinib and Chemotherapy in a Pediatric Patient with Philadelphia-like Acute Lymphoblastic Leukemia with Ebf1-Pdgfrb Fusion Transcript. Leuk. Lymphoma 61 (2), 469–472. 10.1080/10428194.2019.1668938 31558067

[B16] Franca R. Kuzelicki N. K. Sorio C. Toffoletti E. Montecchini O. Poropat A. (2018). Targeting Kinase-Activating Genetic Lesions to Improve Therapy of Pediatric Acute Lymphoblastic Leukemia. Curr. Med. Chem. 25 (24), 2811–2825. 10.2174/0929867324666170727101932 28748759

[B17] Greuber E. K. Smith-Pearson P. Wang J. Pendergast A. M. (2013). Role of ABL Family Kinases in Cancer: from Leukaemia to Solid Tumours. Nat. Rev. Cancer 13 (8), 559–571. 10.1038/nrc3563 23842646 PMC3935732

[B18] Gunnar Cario G. Veronica Leoni V. Valentino Conter V. André Baruchel A. Martin Schrappe M. Andrea Biondi A. (2020). BCR-ABL1-like Acute Lymphoblastic Leukemia in Childhood and Targeted Therapy. Haematologica 105 (9), 2200–2204. 10.3324/haematol.2018.207019 33054045 PMC7556506

[B19] Henriques S. T. Thorstholm L. Huang Y. H. Getz J. A. Daugherty P. S. Craik D. J. (2013). A Novel Quantitative Kinase Assay Using Bacterial Surface Display and Flow Cytometry. PLOS ONE 8 (11), e80474. 10.1371/journal.pone.0080474 24260399 PMC3829888

[B20] Lee S. J. Wang J. Y. (2009). Exploiting the Promiscuity of Imatinib. J. Biol. 8 (3), 30. 10.1186/jbiol134 19435483 PMC2689438

[B21] Lipchik A. M. Perez M. Bolton S. Dumrongprechachan V. Ouellette S. B. Cui W. (2015). KINATEST-ID: a Pipeline to Develop Phosphorylation-dependent Terbium Sensitizing Kinase Assays. J. Am. Chem. Soc. 137 (7), 2484–2494. 10.1021/ja507164a 25689372 PMC4342272

[B22] Matheson E. C. Hall A. G. (2003). Assessment of Mismatch Repair Function in Leukaemic Cell Lines and Blasts from Children with Acute Lymphoblastic Leukaemia. Carcinogenesis 24 (1), 31–38. 10.1093/carcin/24.1.31 12538346

[B24] Ofran Y. Izraeli S. (2017). BCR-ABL (Ph)-like Acute Leukemia-Pathogenesis, Diagnosis and Therapeutic Options. Blood Rev. 31 (2), 11–16. 10.1016/j.blre.2016.09.001 27665024

[B25] Perez M. Blankenhorn J. Murray K. J. Parker L. L. (2019). High-throughput Identification of FLT3 Wild-type and Mutant Kinase Substrate Preferences and Application to Design of Sensitive *In Vitro* Kinase Assay Substrates. Mol. Cel Proteomics 18 (3), 477–489. 10.1074/mcp.RA118.001111 PMC6398213 30541869

[B26] Pisabarro M. T. Serrano L. (1996). Rational Design of Specific High-Affinity Peptide Ligands for the Abl-SH3 Domain. Biochemistry 35 (33), 10634–10640. 10.1021/bi960203t 8718852

[B27] Placzek E. A. Plebanek M. P. Lipchik A. M. Kidd S. R. Parker L. L. (2010). A Peptide Biosensor for Detecting Intracellular Abl Kinase Activity Using Matrix-Assisted Laser Desorption/ionization Time-Of-Flight Mass Spectrometry. Anal. Biochem. 397 (1), 73–78. 10.1016/j.ab.2009.09.048 19818327 PMC2808441

[B28] Plattner R. Irvin B. J. Guo S. Blackburn K. Kazlauskas A. Abraham R. T. (2003). A New Link between the C-Abl Tyrosine Kinase and Phosphoinositide Signalling through PLC-Gamma1. Nat. Cel. Biol. 5 (4), 309–319. 10.1038/ncb949 12652307

[B29] Plattner R. Koleske A. J. Kazlauskas A. Pendergast A. M. (2004). M005401200, Pendergast, A.M Bidirectional Signaling Links the Abelson Kinases to the Platelet-Derived Growth Factor Receptor. Mol. Cel. Biol. 24 (6), 2573–2583. 10.1128/mcb.24.6.2573-2583.2004 PMC355852 14993293

[B30] Quintás-Cardama A. Cortes J. (2009). Molecular Biology of Bcr-Abl1-Positive Chronic Myeloid Leukemia. Blood 113 (8), 1619–1630. 10.1182/blood-2008-03-144790 18827185 PMC3952549

[B31] Quintás-Cardama A. Vaddi K. Liu P. Manshouri T. Li J. Scherle P. A. (2010). Preclinical Characterization of the Selective JAK1/2 Inhibitor INCB018424: Therapeutic Implications for the Treatment of Myeloproliferative Neoplasms. Blood 115 (15), 3109–3117. 10.1182/blood-2009-04-214957 20130243 PMC3953826

[B32] Rix U. Hantschel O. Dürnberger G. Remsing Rix L. L. Planyavsky M. Fernbach N. V. (2007). Chemical Proteomic Profiles of the BCR-ABL Inhibitors Imatinib, Nilotinib, and Dasatinib Reveal Novel Kinase and Nonkinase Targets. Blood 110 (12), 4055–4063. 10.1182/blood-2007-07-102061 17720881

[B33] Roberts K. G. Li Y. Payne-Turner D. Harvey R. C. Yang Y. L. Pei D. (2014). Targetable Kinase-Activating Lesions in Ph-like Acute Lymphoblastic Leukemia. N. Engl. J. Med. 371 (11), 1005–1015. 10.1056/NEJMoa1403088 25207766 PMC4191900

[B34] Roberts K. G. Morin R. D. Zhang J. Hirst M. Zhao Y. Su X. (2012). Genetic Alterations Activating Kinase and Cytokine Receptor Signaling in High-Risk Acute Lymphoblastic Leukemia. Cancer Cell. 22 (2), 153–166. 10.1016/j.ccr.2012.06.005 22897847 PMC3422513

[B35] Roberts K. G. Pei D. Campana D. Payne-Turner D. Li Y. Cheng C. (2014). Outcomes of Children with BCR-ABL1–like Acute Lymphoblastic Leukemia Treated with Risk-Directed Therapy Based on the Levels of Minimal Residual Disease. J. Clin. Oncol. 32 (27), 3012–3020. 10.1200/JCO.2014.55.4105 25049327 PMC4162497

[B36] Roberts K. G. Yang Y. L. Payne-Turner D. Lin W. Files J. K. Dickerson K. (2017). Oncogenic Role and Therapeutic Targeting of ABL-Class and JAK-STAT Activating Kinase Alterations in Ph-like ALL. Blood Adv. 1 (20), 1657–1671. 10.1182/bloodadvances.2017011296 29296813 PMC5728345

[B37] Sjöblom T. Boureux A. Rönnstrand L. Heldin C. H. Ghysdael J. Ostman A. (1999). Characterization of the Chronic Myelomonocytic Leukemia Associated TEL-PDGF Beta R Fusion Protein. Oncogene 18 (50), 7055–7062. 10.1038/sj.onc.1203190 10597306

[B38] Songyang Z. Blechner S. Hoagland N. Hoekstra M. F. Piwnica-Worms H. Cantley L. C. (1994). Use of an Oriented Peptide Library to Determine the Optimal Substrates of Protein Kinases. Curr. Biol. 4 (11), 973–982. 10.1016/S0960-9822(00)00221-9 7874496

[B39] Songyang Z. Carraway K. L. Eck M. J. Harrison S. C. Feldman R. A. Mohammadi M. (1995). Catalytic Specificity of Protein-Tyrosine Kinases Is Critical for Selective Signalling. Nature 373 (6514), 536–539. 10.1038/373536a0 7845468

[B40] Tanaka Y. Kawazu M. Yasuda T. Tamura M. Hayakawa F. Kojima S. (2018). Transcriptional Activities of DUX4 Fusions in B-Cell Acute Lymphoblastic Leukemia. Haematologica 103 (11), e522–e526. 10.3324/haematol.2017.183152 29773604 PMC6278971

[B41] Tang J. Wang J. Y. Parker L. L. (2012). Detection of Early Abl Kinase Activation after Ionizing Radiation by Using a Peptide Biosensor. Chembiochem 13 (5), 665–673. 10.1002/cbic.201100763 22334513 PMC3429332

[B42] Weston B. W. Hayden M. A. Roberts K. G. Bowyer S. Hsu J. Fedoriw G. (2013). Tyrosine Kinase Inhibitor Therapy Induces Remission in a Patient with Refractory EBF1-PDGFRB-Positive Acute Lymphoblastic Leukemia. J. Clin. Oncol. 31 (25), e413–6. 10.1200/JCO.2012.47.6770 23835704

[B43] Wlodarska I. Aventín A. Inglés-Esteve J. Falzetti D. Criel A. Cassiman J. J. (1997). A New Subtype of Pre-B Acute Lymphoblastic Leukemia with T(5;12)(q31q33;p12), Molecularly and Cytogenetically Distinct from T(5;12) in Chronic Myelomonocytic Leukemia. Blood 89 (5), 1716–1722. 10.1182/blood.V89.5.1716 9057655

[B44] Wu D. Sylvester J. E. Parker L. L. Zhou G. Kron S. J. (2010). Peptide Reporters of Kinase Activity in Whole Cell Lysates. Biopolymers 94 (4), 475–486. 10.1002/bip.21401 20593469 PMC2914461

[B45] Yang T. Y. Eissler C. L. Hall M. C. Parker L. L. (2013). A Multiple Reaction Monitoring (MRM) Method to Detect Bcr-Abl Kinase Activity in CML Using a Peptide Biosensor. PLOS ONE 8 (2), e56627. 10.1371/journal.pone.0056627 23437189 PMC3577862

[B46] Yasuda T. Tsuzuki S. Kawazu M. Hayakawa F. Kojima S. Ueno T. (2016). Recurrent DUX4 Fusions in B Cell Acute Lymphoblastic Leukemia of Adolescents and Young Adults. Nat. Genet. 48 (5), 569–574. 10.1038/ng.3535 27019113

[B47] Zhou M. H. Yang Q. M. (2014). NUP214 Fusion Genes in Acute Leukemia (Review). Oncol. Lett. 8 (3), 959–962. 10.3892/ol.2014.2263 25120641 PMC4114590

