# Supplementary material for: A Novel ELISA-Based Peptide Biosensor Assay for Screening ABL1 Activity in vitro: A Challenge for Precision Therapy in BCR-ABL1 and BCR-ABL1 Like Leukemias
Source: Front Pharmacol. 2021 Nov 19;12:749361. doi: 10.3389/fphar.2021.749361 (PMC8640483; doi:10.3389/fphar.2021.749361)
Supplement: Supplementary file 1 [file Presentation1.pdf]

Supplementary Material

Supplementary Materials:

MTT assay

The effect of tyrosine kinase inhibitors (TKI) on cell lines (NALM6, ALL-SIL, K562, REH, HEL, SET2) was determined using the 3-(4,5-dimethylthiazol-2-yl)-2,5-diphenyltetrazolium bromide (MTT) assay. Cells were seeded at 12000 cells/well (60000 cells/ml) in a final volume of 200 µl in 96-well plates. Dilution series of TKI were prepared in culture medium with a range of concentrations of 0.014 - 10 µM for imatinib and 0.016 - 10 µM for ruxolitinib. The experiment was repeated three times for imatinib and two times for ruxolitinib, plus each condition was seeded in duplicate. Plates were incubated for 72 hours at 37 °C; 20 µl MTT 5 mg/ml (M2128-1G Sigma-Aldrich, Italy) were added in each well four hours before the end of the incubation and the absorbance was measured with a spectrophotometer ((570-630 nm wavelength) Microplate Reader EL311 (214891, BioTek Instruments, Inc).

Trypan blue exclusion assay

B-ALL cells viability in presence of TKI was determined by trypan blue exclusion assay. Cells were seeded at 12000 cells/well (60000 cells/ml) in a final volume of 200 µl in 96-well plates, after exposure to imatinib (0.38, 5.5 and 10 µM) and ruxolitinib (10 and 50 µM). Number of viable cells was manually counted by a single operator on aliquots of cell suspensions added with 0.04% trypan blue (T8154, Sigma-Aldrich, Italy) loaded into a Bürker counting chamber (BR718920 Sigma-Aldrich, Italy). Double counting was done for each condition and cells that stained blue were considered as nonviable. The experiment was repeated five times for imatinib and two times for ruxolitinib.

Cell lysates preparation

Cell protein lysates were prepared for both western blot and PABL-based ELISA assays. The method of extraction has been optimized by Professor Sorio (University of Verona) and requires the use of kinases and phosphatases inhibitors to maintain the integrity of BCR-ABL1 in K562 cell line. Twenty million cells were lysed in ice with 150 µl of lysis buffer containing 50 mM Tris-HCl (pH 7.4), 1% Triton-X, 150 mM NaCl, 2 mM EDTA, with addition of LeukoProtect lysis buffer (AB Analitica, Padova, Italy, sodium orthovanadate (Na3VO4) 100 µM (S6508, Sigma-Aldrich, Italy), sodium fluoride (NaF) 10 mM (450022, Sigma-Aldrich, Italy) and DTT 1 mM (43819, Sigma-Aldrich, Italy), 1x PhosSTOP tablets (4906845001, Sigma-Aldrich/Merk, Italy). The lysis procedure consists of pipetting 20 times the cell suspension, vortexing and centrifuging at 4 °C for 30 min at 13000 g. The collected supernatant was rapidly frozen in liquid nitrogen and stored at -80 °C in cryovials. Finally protein concentrations were determined by Bradford analysis (B6916, Sigma-Aldrich, Italy) and

absorbance was measured at 570 nm on a Microplate Reader EL311 (214891, BioTek Instruments, Inc).

Western Blot

Whole cell extracts (25 µg) were fractionated by SDS-PAGE using 10% Bolt™ 10% Bis-Tris Plus Gels 12-well (NW00102BOX, Thermo Fisher Scientific, Italy) or NuPAGETM 3 to 8%, Tris-Acetate (EA03752BOX, Thermo Fisher Scientific, Italy). Prestained protein ladder (LC5925- Thermofisher) was used. Proteins were transferred to nitrocellulose membranes (PB7320, Thermo Fischer Scientific, Italy) using Electrophoresis Power Supply (EPS301, Thermo Fischer Scientific, Italy). After incubation with 5% nonfat milk (non-phospho-protein) or 5% BSA (phospho-protein) in Tris buffered saline (50 mM Tris-Cl, 150 mM NaCl, pH 7,5) with 0.1% Tween-20 (T-TBS) for 1 hour, the membranes were incubated with antibodies against: PDGFRB (1:5000 in T-TBS 5% nonfat milk, ab32570, Abcam), ABL1 (1:1000 in T-TBS 5% nonfat milk, #2862, Cell Signaling Technology), phospho-ABL1 (Y245) (1:1000 in T-TBS 5% BSA, #2861, Cell Signaling Technology), actin (1:3000 in T-TBS 5% nonfat milk, ab218787, Abcam) at 4 °C, overnight. Membranes were washed and incubated 1 hour at 4 °C with a 1:10000 dilution in T-TBS 5% nonfat milk of horseradish peroxidase-conjugated anti-rabbit antibodies (AP132P, Merk). PDGFRB blots were developed with LiteAblo TURBO Extra Sensitive Chemiluminescent Substrate (EMP012001 Euroclone), GBX Developer/Replenisher (P7042, Sigma-Aldrich, Italy) and GBX Fixer/Replenisher (P7167, Sigma-Aldrich, Italy). Exposure of membrane was made on light-sensitive film (Z373508- Carestream® BioMax® light film, Sigma-Aldrich). ABL1 blots were developed with LiteAblo TURBO Extra Sensitive Chemiluminescent Substrate and detection was performed using ChemiDoc Imaging System (Bio-Rad Laboratories). For each antibody, experiments were repeated twice with similar results and bands of interest were quantified with ImageJ software, after normalizing with actin.

1 Supplementary Figures and Tables

For more information on Supplementary Material and for details on the different file types accepted, please see here. Figures, tables, and images will be published under a Creative Commons CC-BY licence and permission must be obtained for use of copyrighted material from other sources (including re-published/adapted/modified/partial figures and images from the internet). It is the responsibility of the authors to acquire the licenses, to follow any citation instructions requested by third-party rights holders, and cover any supplementary charges.

Supplementary Figures:

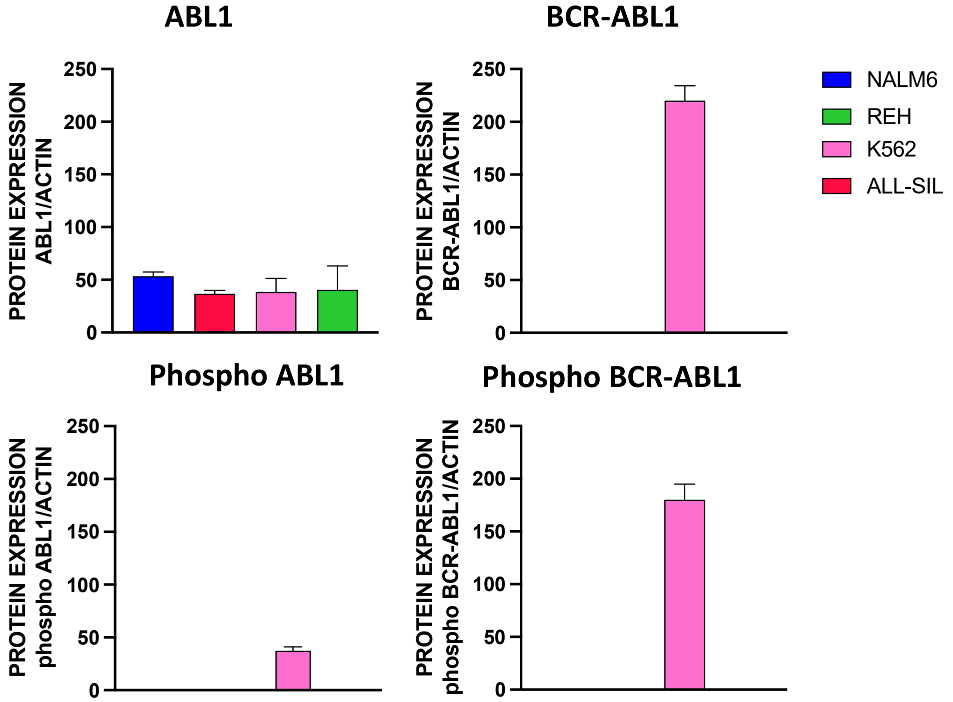

Figure S1. Proteins quantification by ImageJ after western blot analysis (n=2).

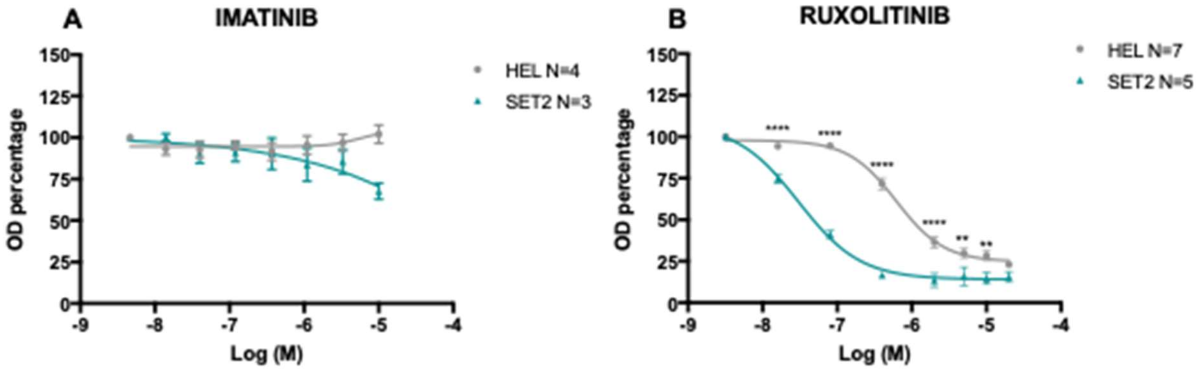

Figure S2. Dose-response curve of HEL and SET2 with A) imatinib and B) ruxolitinib. Cell lines were seeded at 12000 cells/well with a range of concentrations of 0.014 - 10 µM for imatinib and 0.016 - 10 µM for ruxolitinib. MTT assay was performed after 72 h of incubation. Error bars represent mean ± SEM. HEL *versus* SET2: **, <0.01; ***, <0.001; ****, <0.0001, two-way ANOVA, Bonferroni post-test.

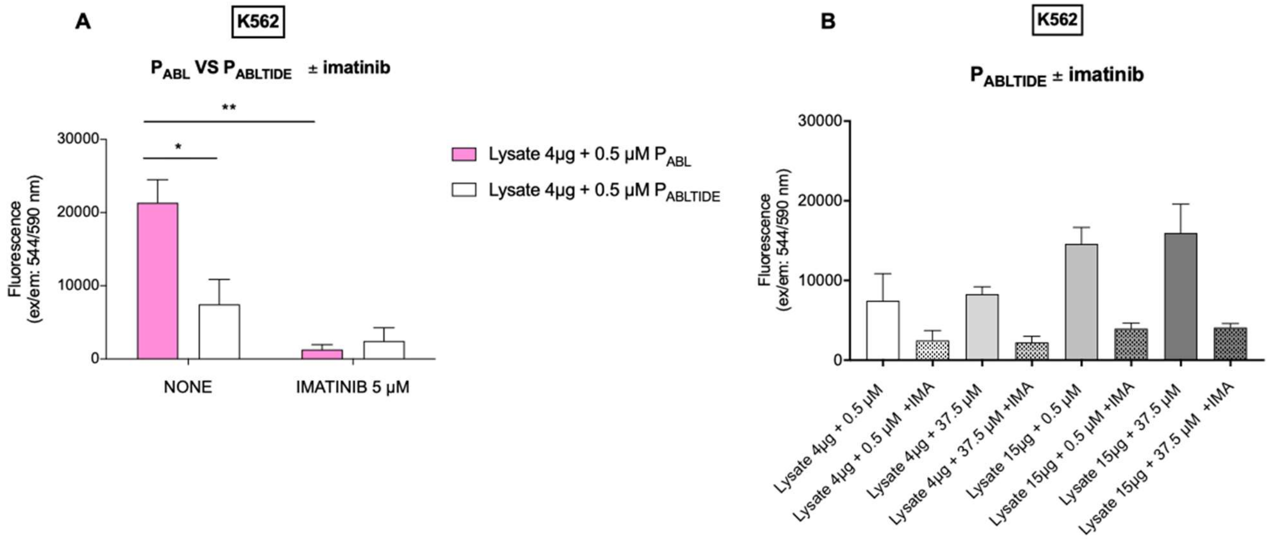

Figure S3. $P_{ABLTIDE}$-based ELISA assay in the presence and absence of 5 µM imatinib. A) Comparison between P_{ABL} and $P_{ABLTIDE}$ phosphorylation levels; B) $P_{ABLTIDE}$ phosphorylation levels incubated with different amount of lysates and probe. The graphs show data obtained from 2 independent experiments. Fluorescence values in ordinate. P-value calculated according two-way ANOVA, Bonferroni post-test: * = 0.023; ** = 0.0027). IMA: imatinib.

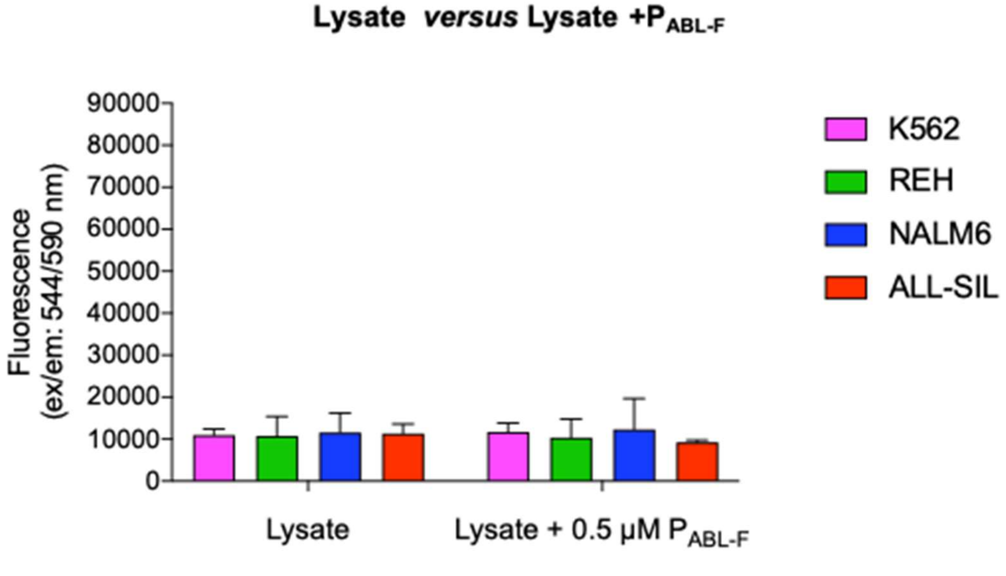

Figure S4. P_{ABL-F}-based ELISA assay. The graph shows data obtained from 4 independent experiments. Fluorescence values in ordinate. There is no significant increase in terms of phosphorylation levels for all the lines after incubation with the peptide P_{ABL-F} (two-way ANOVA Bonferroni post-test for multiple comparison).

Supplementary Tables:

IMATINIB	K562	NALM6	ALL-SIL	REH
**None**	100 %	100 %	100 %	100 %
**0.38 μM**	37.53 ± 9.11 %	81.52 ± 20.47 %	29.30 ± 12.86 %	120.78 ± 9.18 %
**5.5 μM**	20.65 ± 3.73 %	51.17 ± 9.93 %	20.94 ± 1.61 %	107.79 ± 2.45 %
**10 μM**	15.38 ± 5.35 %	38.73 ± 10.67 %	18.58 ± 3.59 %	74.68 ± 31.53 %

RUXOLITINIB	K562	NALM6	ALL-SIL	REH
**None**	100 %	100 %	100 %	100 %
**10 μM**	63.69 ± 16.48 %	65.17 ± 32.62 %	56.57 ± 11.73 %	72.79 ± 3.12 %
**50 μM**	17.26 ± 8.53 %	18.70 ± 7.04 %	23.86 ± 4.48 %	38.33 ± 20.93 %

Table S1. Cells viability in presence of TKI according to trypan blue exclusion assay (mean±SD, N=5 imatinib, N=2 ruxolitinib).

	LYSATE	LYSATE + P_{ABL}	P_{ABL} + imatinib 5 μM	P_{ABL} + ruxolitinib 52 nM	P_{ABL}+ ruxolitinib 5 μM
**K562**	9.35 ± 2.35 % N=12	41.43 ± 3.45 % N=12	12.17 ± 2.83% N=12	39.41 ± 3.31 % N=6	41.08 ± 3.20 % N=3
**REH**	5.10 ± 0.88 % N=12	28.40 ± 3.50 % N=12	14.47 ± 3.05 % N=12	29.18 ± 5.97% N=6	17.42 ± 3.14 % N=3
**NALM6**	6.93 ± 1.53 % N=12	28.16 ± 3.23 % N=12	6.57 ± 1.62% N=12	28.02 ± 7.54 % N=6	15.43 ± 8.30 % N=3
**ALL-SIL**	5.96 ± 1.09 % N=7	31.76 ± 2.79 % N=7	10.40 ± 1.95 % N=7	31.02 ± 4.13% N=6	18.08 ± 6.12 % N=3

Table S2. Phosphorylation of P_{ABL} biosensor is expressed as a percentage relative to the fully phosphorylated $P_{PHOSPHO\text{-}ABL}$, used as reference.

	LYSATE (N=4)	LYSATE + $P_{ABL\text{-}F}$ (N=4)
**K562**	4.60 ± 0.92 %	6.24 ± 0.84%
**REH**	3.94 ± 1.08 %	5.72 ± 2.01%
**NALM6**	3.89 ± 1.08 %	5.57 ± 2.40%
**ALL-SIL**	6.11 ± 0.60 %	4.97 ± 0.14%

Table S3. Phosphorylation is expressed as a percentage related to $P_{PHOSPHO\text{-}ABL}$, from 4 different lysates.
